# Glycemic control and its associated factors among adult diabetic patients in Southern Ethiopia: a cross-sectional study

**DOI:** 10.4314/ahs.v24i1.23

**Published:** 2024-03

**Authors:** Ageze Abose, Aklilu Getachew, Fanta Obsa, Shiferaw Bekele, Kassahun Haile, Selamu Abose

**Affiliations:** 1 Department of Medical Laboratory Science, College of Medicine and Health Science, Wachemo University, Hossana, Ethiopia; 2 School of Medical Laboratory Science, Institute of Health, Jimma University, Jimma, Ethiopia; 3 Department of Medical Laboratory Science, College of Medicine and Health Science, Wolkite University, Wolkite, Ethiopia; 4 Department of midwifery, College of Medicine and Health Science, Wachemo University, Hossana, Ethiopia

**Keywords:** Diabetes mellitus, Glycemic control, Glycated hemoglobin, Hossana, Ethiopia

## Abstract

**Background:**

Diabetes mellitus is a group of common metabolic disorders that share the phenotype of hyperglycemia. Chronic hyperglycemia causes vascular complications, mortality, and life-threatening disabilities in low-income countries including Ethiopia. Glycemic control status in diabetic patients is crucial to maintain the blood glucose level at the optimal level and to reduce the risk of diabetes-related complications and mortality. However, there is limited data on poor glycemic control status and its associated factors among diabetic patients in southern Ethiopia, particularly in the study area. Thus, this study aimed to determine glycemic control status and its associated factors using glycated hemoglobin among adult diabetic patients at Nigist Elleni Mohammad Memorial Referral Hospital, Hossana, southern Ethiopia.

**Materials and methods:**

A facility-based cross-sectional study was conducted from May 1 to June 30, 2020. A systematic random sampling technique was used to recruit 307 diabetic patients at follow-up. Interviewer administered questionnaire was used to collect data on sociodemographic, clinical, and behavioral characteristics. Five milliliters of venous blood samples were collected to determine lipid profiles and hemoglobin A1C. Lipid profiles and hemoglobin A1C were measured by Cobas c311 analyzer. The data were analyzed by SPSS version 20. Bivariable and multivariable logistic regression were used to determine associated factors with poor glycemic control status. P-value <0.05 was considered statistically significant.

**Result:**

The overall prevalence of poor glycemic control among the study participants based on hemoglobin A1C ≥7% was 82.4%. Having a history of diabetic complications (AOR: 7.09, 95%CI: 1.72–29.16), duration of diabetes ≥7 years (AOR: 4.09, 95%CI: 1.38–12.08), insulin and oral hypoglycemic agents (AOR: 0.106 95%CI: 0.02–0.44), lack of self-glucose monitoring (AOR: 8.27, 95%CI: 1.61–42.46), lack of physical exercise (AOR: 5.5, 95%CI: 1.6–18.9) and dyslipidemia (AOR: 2.74, 95%CI: 1.12–6.66) were significantly associated with poor glycemic control.

**Conclusion:**

A high prevalence of poor glycemic control status (82.4%) was observed among diabetic patients in this study area, and disease-related factors like duration of diabetes, complication, treatment type and lack of self-glucose monitoring, physical exercise, and dyslipidemia were identified as factors significantly associated with poor glycemic control status. The finding of the current study should be taken into account to conduct a strategic and timely intervention on significantly associated factors to delay diabetic complications and to improve the health outcome of diabetic patients. Routine screening and monitoring of dyslipidemia and providing health education on behavioral factors were the necessary measures that should be conducted to reduce the burden of poor glycemic control status among diabetic patients.

## Introduction

Diabetes mellitus (DM) is a group of common metabolic disorders that share the phenotype of hyperglycemia, which is caused by the demise of insulin secretion, insulin action, or both 1-3. Persistent chronic hyperglycemia causes vascular complications among diabetic patients due to non-enzymatic glycation of proteins, production of oxida-tive stress, and secretion of various cytokines which enhance inflammatory response 4-6.

Diabetes mellitus is a global health problem. According to the International Diabetes Federation (IDF) report in 2019, globally 463 million people were affected by diabetes mellitus from this 79.4% were found in low and middle-income countries. Currently, Ethiopia has been challenged by the growing magnitude of non-communicable diseases such as diabetes mellitus. International Diabetes Federation reported 1.7 million people aged between 20-79 years were affected in Ethiopia in 2019, and another study reported a 3.2% national burden of diabetes in age 20-70 years 7.

Glycemic control is fundamental to diabetes management, maintaining the blood glucose level at the optimal level, and reducing the risk of diabetes-related complications and mortality 3. It is primarily assessed with the hemoglobin A1C test. Hemoglobin A1C is glycated hemoglobin and formed by a non-enzymatic reaction of glucose with the N-terminal amino group of the beta-chain of normal adult HgbA. It is a standard test to determine the glycemic control status of diabetic patients 1,10. However, glycemic control status in most of the developing countries including Ethiopia was majorly assessed by fasting blood glucose which reflects on spot status, which compromises the quality of a patient's life.

Poor glycemic control is the major problem that causes blindness, kidney failure, lower limb amputation, and several other long-term consequences that impact significantly the quality of life. The long-term complications that result from poor glycemic control contribute substantially to the morbidity, mortality, and economic burden of diabetes 8,9 Due to the complex nature of the disease and its linkage with multifactor, the majority of diabetic patients fail to achieve an adequate level of glycemic control 11,12. Globally the burden of poor glycemic control is increasing at an alarming rate, studies revealed 91.8%,74.9%, 82%, 76%, 76.7%, and 80% prevalence of poor glycemic control in India 13, Tabuk, Saudi Arabia^14^, Bangladesh^11^, Venezuela^15^, Asmara,Eritrea^16^ and at Tikur Anbessa Specialized Hospital, Ethiopia^17^, respectively. Another study conducted in Ethiopia at the University of Gondar Referral Hospital among diabetic patients revealed poor glycemic control was higher among Type 1 diabetic patients (82.9%) as compared with Type 2 patients (57.5%) 18.

In recent years, non-communicable diseases became a major health problem in middle and low-income countries that resulting in death, disabilities, and huge economic loss. Diabetes is one of the leading non-communicable diseases which is increasing at an alarming rate and a lifelong risk factor for chronic complications. Determination of glycemic control status and its associated factors among diabetic patients with appropriate methods is important for the management of diabetic patients, improvements in the quality of patient's life, and design of intervention mechanisms to reduce complications, mortality, and related disabilities. There is a paucity of data on poor glycemic control and its associated factor among diabetic patients in southern Ethiopia, particularly in the study area, and in addition, most of the previous studies conducted in Ethiopia was performed by fasting blood glucose level which reflects on spot status and unable to measure the exact burden of the poor glycemic status. The major tool to measure glycemic control is glycated hemoglobin, but most of the previous studies in Ethiopia are not performed with this standard test. Therefore, this study aimed to assess glycemic control and its associated factor by using glycated hemoglobin among diabetic patients in southern Ethiopia.

## Materials and methods

### Study area

This study was conducted at Wachemo University Nigist Elleni Mohammed Memorial Referral Hospital (WUNEMMRH) which is located in Hossana town, Hadiya Zone, Southern Ethiopia. The town is 232 km far from the capital city of Ethiopia, Addis Ababa. The hospital provides services to around 3.2 million catchment area population 19. The study was conducted on adult diabetic patients attending their follow-up at the chronic care clinic of WUNEMMRH.

### Study design and period

A hospital-based cross-sectional study design was carried out among adult diabetic patients from May 2020 to June 2020.

### Sample size determination and sampling technique

The sample size was determined by using a single population proportion formula by considering a 95% level of confidence interval, 5% margin of error, and 59.4% prevalence of poor glycemic control from the study conducted in Jimma University Specialized Hospital 20. After using the finite population correction formula and adding 10% of non–the response rate we got the final sample size of 320. A systematic random sampling technique was used to select study participants from diabetic patients attending chronic care follow-up clinics. Within similar study periods in the last year, 1230 diabetic patients were attending their follow-up at the chronic care clinic of WUNEMMRH. When we divide the total population by sample size (1230/320) we got the interval size(k) value of around 4. Therefore, we randomly select number 3 from 1-4, and then we start with number 3 and took every 4th study participant until attending the final sample size.

### Inclusion and exclusion criteria

Patients who had a regular follow-up were on treatment for at least 3 months and aged ≥18 years were included in this study. Patients who were pregnant, newly diagnosed, and severely ill were excluded.

### Data collection techniques and laboratory procedures

Data on socio-demographic, behavioral, and clinical characteristics were collected by trained nurses via a structured questionnaire. Data on socio-demographic characteristics included age of patients, sex, occupation, educational status, marital status, residence, and monthly income. Data on clinical and self-monitoring behavior included type of DM, type of medication, history of complication, duration of diabetes from time of diagnosis, family history, hypertension as co-morbidity, alcohol drinking, physical exercise, and self-glucose monitoring. Type of DM, type of medication, history of complication, hypertension as co-morbidity were collected by medical chart review, and duration of diabetes from time of diagnosis and family history were collected by face-to-face interview. Medication adherence was assessed by a modified eight-item Morisky medication adherence scale, based on this scale patient's response can be classified into three main parts these are poor medication adherence, moderate medication adherence, and good medication adherence 21.

Data on anthropometrical measurements were also measured. The Weight and height of study participants were measured by using calibrated FAZZINI scale S7200HR, Vidmodrone (MI), Italy with height rodas the patients wore light clothes and no shoes. Body mass index (BMI) was calculated from the body weight (kg) and height (m) by dividing weight by height square 22,23.

For the laboratory data, 5ml of blood was withdrawn from each study participant after overnight fasting. Three ml of blood was collected in a gel separator tube to determine the levels of lipid profiles (TC, HDL-C, LDL-C, TG), and 2ml of blood was collected in an EDTA tube to determine hemoglobin A1C by qualified laboratory professionals. Cobas 4000 series Cobas c311 (Basel, Switzerland) analyzer was used to determine lipid profile and hemoglobin A1C level.

An enzymatic colorimetric test method was used to determine total cholesterol in human serum. Cholesterol esters were broken down by cholesterol esterase to release free cholesterol and fatty acids then cholesterol was oxidized by cholesterol oxidase to cholest-4-en-3-one and hydrogen peroxide. In the presence of peroxidase, hydrogen peroxide coupled with phenol and 4-aminophenazone to form a red quinoneimine dye. The color intensity of the dye formed indirectly proportional to the cholesterol concentration.

The enzymatic method was used to determine triglycerides. Triglycerides are hydrolyzed by lipoprotein lipase to glycerol and fatty acids. Glycerol is phosphorylated to glycerol-3-phosphate in ATP requiring a reaction by glycerol kinase. Glycerol-3-phosphate is oxidized by glycerol phosphate oxidase to form dihydroxyacetone phosphate and hydrogen peroxide. In the presence of peroxidase, hydrogen peroxide affects the oxidative coupling of 4-chlorophenol and 4-aminophenazone to form a red-colored dye. The increase in absorbance is directly proportional to the concentration of triglycerides in the serum.

A homogeneous enzymatic colorimetric method was used to determine high-density lipoprotein. In the presence of magnesium ions, dextran sulfate selectively forms water-soluble complexes with LDL, VLDL, and chylomicrons which are resistant to PEG-modified enzymes. The cholesterol concentration of HDL-cholesterol is determined enzymatically by cholesterol esterase and cholesterol oxidase coupled with PEG to the amino groups. Cholesterol esters are broken down quantitatively into free cholesterol and fatty acids by cholesterol esterase. In the presence of oxygen, cholesterol is oxidized by cholesterol oxidase to delta 4-cholestenone and hydrogen peroxide. In the presence of peroxidase, the hydrogen peroxide generated reacts with 4-amino-antipyrine and HSDA (Sodium N-(2-hydroxy-3-sulfopropyl)-3, 5-dimethoxyaniline) to form a purple-blue dye. The color intensity of the blue dye formed is directly proportional to the HDL-cholesterol concentration. It is determined by measuring the increase in absorbance photometrically.

The homogeneous enzymatic colorimetric assay was used for the determination of LDL cholesterol. LDL-cholesterol is selectively solubilized by nonionic detergent. The presence of the Mg++a sugar compound markedly reduces the enzymatic reaction of the cholesterol measurement in VLDL and chylomicrons. The combination of a sugar compound with detergent enables the selective determination of LDL-cholesterol in serum. In the presence of cholesterol esterase, colesterol esters quantitatively break down into free cholesterol and fatty acids. In the presence of oxygen, cholesterol is oxidized by cholesterol oxidase to delta 4-cholestenone and hydrogen peroxide. In the presence of peroxidase, the hydrogen peroxide generated reacts with 4-aminoantipyrine and HSDA to form a purple-blue dye. The color intensity of this dye is directly proportional to the cholesterol concentration and is measured photometrically.

The HbA1c determination is based on the turbidimetric inhibition immunoassay (TINIA). This method uses TTAB (Tetradecyltrimethylammonium bromide) as the detergent in the hemolyzing reagent to eliminate interference. Glycated hemoglobin (HbA1c) in the sample reacts with anti-HbA1c antibody to form soluble antigen-antibody complexes. Since a specific HbA1c antibody site is present only once on the HbA1c molecule, the complex formation does not take place. The Polyhapten reagent reacts with excess anti-HbA1c antibodies to form an insoluble antibody-poly hapten complex which can be measured turbid metrically.

### Data quality assurance

To ensure data quality, training was given for data collectors on data collection procedures and standard operating procedures, and the completeness of each questionnaire was checked regularly. Daily maintenance of the clinical chemistry analyzer was done, and all tests were done following standard operating procedures and manufacturer instructions. Quality controls were done for lipid profile and hemoglobin A_1_C tests before running the patient sample. The expiration date for all reagents was checked. Calibrated weight and height measuring instrument was used. Furthermore, data were entered in Epi-data version 3.1 to improve data quality.

### Data analysis

Data were entered into Epi data version 3.1(Epi-Data, Odense, Denmark) and analyzed by using Statistical Package for Social Science (SPSS) version 20 (IBM, Chicago, IL, U.S.A). Data were summarized by tables, graphs, and descriptive statistics. A binary logistic regression analysis model was used to determine the association between poor glycemic control status and independent variables. Bivariate analysis was performed for each independent variable to select candidate variables for multivariable logistic regression. The variables with a p-value <0.25 in bivariate analysis were taken to multivariable logistic regression. The multivariable logistic regression was done to control confounding and identify associated risk factors with poor glycemic control. P-value <0.05 was considered statistically significant and an adjusted odds ratio was used to determine the strength of the association of variables.

### Operational definition

**Dyslipidemia:** is an abnormality in any one of lipid profile when HDL-C < 40 mg/dl, LDL-C >130 mg/dl, cholesterol 200mg/dl and TG>150mg/dl

**Good glycemic control:** HbA1C< 7% 1.

**Good medication adherence:** When the participants score all eight-item Morisky scale questions.

**Moderate medication adherence:** When the study participants respond to 6-7 questions from eight-item Morisky scale questions.

**Poor glycemic control:** HbA1C ≥ 7% 1.

**Poor medication adherence:** When the study participants respond to less than 6 questions from eight items Morisky questions.

### Ethical considerations

Ethical clearance was obtained from Jimma University Ethical Review Board. A letter of cooperation was written to WUNEMMRH and permission was obtained from the hospital administration. Written informed consent was obtained from each study participant after explaining the purpose and procedures of the study. The study participant results were kept confidential and they were assured that only aggregate data will be reported. All necessary results of the participant were communicated with the physician for proper management. This study was conducted in accordance with the Declaration of Helsinki.

## Results

Socio-demographic characteristics of study participants A total of 320 diabetic patients were recruited to participate in this study of which 307(96%) provided complete information. Of 307 diabetic patients, 280 were type 2 and 27 patients were type1. The majority of the study participants were male 201(65.5%). The mean (SD) age of the study participants was 47.44± (13.3) years. About 48.5% of diabetic patients were in the age range of 45-64 years. The majority of the study participants were married 252(82.1%), urban residents 213(69.4%), and monthly income >1000ETB 268(87.3%) (Table 1).

**Table 1:** Socio-demographic characteristics DM patients attending at Wachemo University Nigist Elleni Mohamed memorial referral and teaching hospital, Hossana, Southern Ethiopia, May 1 to June 30, 2020(N=307)

Variables	Catagories	N (%)
Age in years	<25	20 (6.5)
	25-44	102(33.2)
	45-64	149(48.5)
	≥65	36(11.7)
Sex	Male	201(65.5)
	Female	106(34.5)
Educational status	No formal education	66(21.5)
	Primary (1-8)	79(25.7)
	Secondary (9-12)	62(20.2)
	Diploma and above	100(32.6)
Marital status	Single	34(11.1)
	Married	252(82.1)
	Divorced	14(4.6)
	Widowed	7(2.3)
Residence	Urban	213(69.4)
	Rural	94(30.6)
Occupation	Government employed	95(30.9)
	Housewife	65(21.2)
	Merchant	69(22.5)
	Farmer	53(17.3)
	Other	25(8.1)
Monthly income	≤1000ETB	30(9.8)
	>1000ETB	268(87.3)
Type of DM	Type1	27(8.8)
	Type2	280(91.2)

**Note:** ETB: Ethiopian Birr, DM: diabetes mellitus, other (NGO Worker, student, and private worker).

### Clinical and behavioral characteristics

Of the study participants, 87(28.3%) had a history of diabetic complications. The majority of 289(94.1%) and 294(95.8%) study participants didn't perform regular physical exercise and self-glucose monitoring. According to the duration of diabetes from the time of diagnosis, 213(69.4%) were less than seven years and 94(30.6%) were greater than or equal to seven years. Family history of diabetes was observed in 54(17.6%) and hypertension a co-morbidity was reported by 58(18.9%) study participants. Most of the patients 193(62.9%) were treated with oral hypoglycemic agents only. Ninety-six (31.3%) patients were treated with insulin only and 18(5.9%) were treated with a combination of oral hypoglycemic agents with insulin. Among the study participants 164(53.4%), 90(29.3%), and 53(17.3%) had good, moderate, and poor medication adherence, respectively. Out of the study participants, 266(86.6%) had dyslipidemia (Table 2).

**Table 2:** Clinical and behavioral characteristics of DM patients attending at Wachemo University Nigist Elleni Mohammed Memorial Referral and Teaching Hospital, Hossana, Southern Ethiopia, May 1 to June 30, 2020

Variables	Catagories	N (%)	Glycemic control	
			Good (%)	Poor(%)
History of complication	Yes	87(28.3)	3(3.4)	84(96.6)
	No	220(71.7)	51(23.2)	169(76.8)
Physical Exercise	Yes	18(5.9)	11(61.1)	7(38.9)
	No	289(94.1)	43(14.9)	246(85.1)
Self-glucose monitoring	Yes	13(4.2)	9(69.2)	4(30.8)
	No	294(95.8)	45(15.3)	249(84.7)
Alcohol drinking	Yes	8(2.6)	1(12.5)	7(87.5)
	No	299(97.4)	53(17.7)	246(81.3)
Duration of DM	<7 Years	213(69.4)	47(22)	166(78)
	≥7 Years	94(30.6)	7(7.4)	87(92.6)
Family history of DM	Yes	54(17.6)	10(18.5)	44(81.5)
	No	253(82.4)	44(17.4)	209(82.6)
Hypertension as comorbidity	Yes	58(18.9)	6(10.3)	52(89.7)
	No	249(81.1)	48(19.3)	201(81.7)
Type of medication	Insulin only	96(31.3)	13(13.5)	83(86.5)
	OHA	193(62.9)	30(15.5)	163(84.5)
	Insulin and OHA	18(5.9)	11(61.1)	7(39.9)
Medication Adherence	Good	164(53.4)	36(22)	128(78)
	Moderate	90(29.3)	12(13.3)	78(86.7)
	Poor	53(17.3)	6(11.3)	47(88.7)
BMI	Under weight	15(4.9)	2(13.3)	13((86.7)
	Normal	145(47.2)	26(17.9)	119(82.1)
	Over weight	117(38.1)	22(18.6)	95(81.4)
	Obesity	30(9.8)	4(13.3)	26(86.7)
Dyslipidemia	Yes	266(86.6%)	41(15.4)	225(84.6)
	No	41(13.4)	13(31.7)	28(68.3)

### Prevalence of poor glycemic control among study participants

**Figure1:**
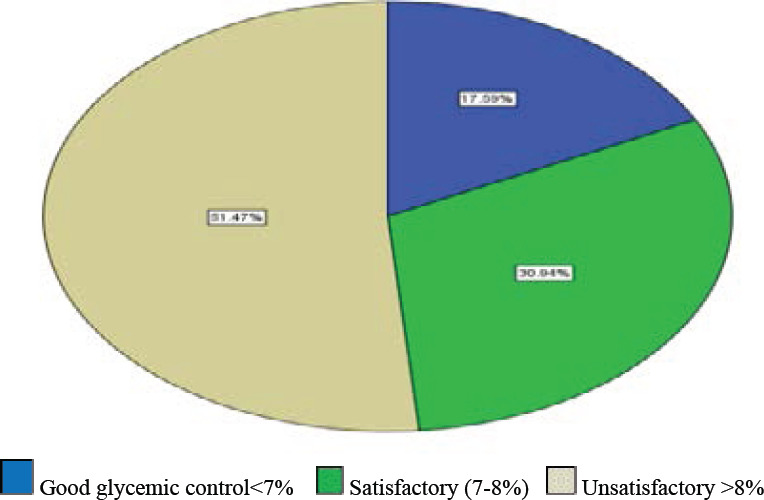
Degree of glycemic control status among study participants, HbA1c < 7% is good glycemic control, and HbA1c ≥7% is poor glycemic control which includes satisfactory and unsatisfactory glycemic control at Wachemo University Nigist Elleni Mohammed Memorial Referral Hospital, 2020

The mean and standard deviation value of hemoglobin A1C level was 8.77±2.32%. The overall prevalence of poor glycemic control with hemoglobin A1C ≥ 7% was 253 (82.4%). Of the study participants, 17.6%, 30.9%, and 51.5% had good, satisfactory, and unsatisfactory glycemic control status, respectively (Figure 1). Poor glycemic control was predominant among 45-64 age groups 127(85.2%), marrie211(83.7%), rural residents 80 (85.1%), participants who had a history of diabetic complication 84(96.6%), diabetic duration ≥7 years 87(92.6%), poor medication adherence 47(88.7%) and dyslipidemic 225 (84.6) (Table2).

### Factors associated with poor glycemic control

After adjusting for other variables; having a history of diabetic complications, duration of diabetes ≥7 years, lack of self-glucose monitoring, lack of regular physical exercise, dyslipidemia, insulin, and the oral hypoglycemic agent was significantly associated with poor glycemic control (Table3). Study participants who had a history of diabetic complications were 7 times more likely poor glycemic status than their counterparts (AOR: 7.093, 95% CI: 1.725-29.163). Diabetic patients who had a diabetic duration of ≥7 years were higher odds of poor glycemic status than the duration of diabetes less than 7 years (AOR: 4.096, 95%CI: 1.388-12.089). Study participants who lack self-glucose monitoring were 8 times higher odds of poor glycemic status than their counterparts (AOR: 8.276, 95%CI: 1.613-42.461). Among the study participants, a significantly high prevalence of poor glycemic status was observed in dyslipidemic, physically inactive, and diabetic patients who took insulin and oral hypoglycemic agent (Table3).

**Table 3:** Multivariable logistic regression analysis of factors associated with poor glycemic control among DM patients attending at Wachemo University Nigist Elleni Mohammed Memorial Referral and Teaching Hospital, Hossana, Southern, Ethiopia, May 1 to June30.2020

Variables	Catagories	Glycemic control status		COR (95%CI)	AOR (95% CI)
		Good (%)	Poor(%)		
Educational status	No formal education	7(10.6)	59(89.4)	3.117(1.268-7.662)	2.645 (0.913-7.66)
	Primary	14(17.7)	65(82.3)	1.717(0.83-3.552)	1.503 (0.601-3.76)
	Secondary	6(9.7)	56(90.3)	3.452(1.334-8.932)	3.14(1.003-9.84)
	Diploma & above	27(27)	73(73)	Ref	Ref
History of complication	Yes	3(3.4)	84(96.6)	8.45(2.562-27.87)	7.093 (1.725-29.16)*
	No	51(23.2)	169(76.8)	Ref	Ref
Physical Exercise	Yes	11(61.1)	7(38.9)	Ref	Ref
	No	43(14.9)	246(85.1)	8.99 (3.302-24.47)	5.503 (1.601-18.9)*
Self-glucose Monitoring	Yes	9(69.2)	4(30.8)	Ref	Ref
	No	45(15.3)	249(84.7)	12.45(3.676-42.2)	8.276 (1.613-42.5)*
Duration of DM	<7 Years	47(22)	166(78)	Ref	Ref
	≥7 Years	7(7.4)	87(92.6)	3.519(1.526-8.11)	4.096 (1.388-12.1)*
Hypertension as co morbidity	Yes	6(10.3)	52(89.7)	2.07 (0.84-5.1)	1.379 (0.448-4.24)
	No	48(19.3)	201(81.7)	Ref	Ref
Type of medication	Insulin only	13(13.5)	83(86.5)	10.03 (3.295-30.55)	Ref
	OHA	30(15.5)	163(84.5)	8.54 (3.065-23.78)	1.16 (0.534-2.52)
	Insulin & OHA	11(61.1)	7(39.9)	Ref	0.106 (0.025-0.444)*
Medication Adherence	Good	36(22)	128(78)	Ref	Ref
	Moderate	12(13.3)	78(86.7)	1.649 (0.839-3.24)	1.241 (0.559-2.76)
	Poor	6(11.3)	47(88.7)	2.611 (0.874-7.798)	2.131(0.546-8.32)
Dyslipidemia	Yes	41(15.4)	225(84.6)	2.548(1.219-5.325)	2.743(1.129-6.662)*
	No	13(31.7)	28(68.3)	Ref	Ref

**Note:** *: significant CI in a multivariable model, Ref: reference, OHA: oral hypoglycemic agents, AOR: adjusted odds ratio, CI: confidence interval

## Discussion

The current study attempted to determine the prevalence of glycemic control and its associated factor using glycated hemoglobin among diabetic patients in southern Ethiopia. The overall prevalence of poor glycemic control in the current study among diabetic patients was 82.4%. This finding was in line with a previous studies conducted in Bangladesh (82%) 11, India (76.6%) 13, Asmara, Eritrea (76.7%) 16, and Addis Ababa, Ethiopia (80%) 17. Our finding was higher than studies conducted across Europe (37.4%) 24, Tanzania (69.7%) 25, Nekemte Referral Hospital (64.9%) 26, and Gondar (60.5%) 27. However, lower than study finding reported from India (91.8%) 28 and South Africa (86.5%) 29. Variation in the burden of the poor glycemic status might be due to the difference in the method of glucose measurement, sample size, and cut-off value.

Participants who had a history of diabetic complications were higher odds of having poor glycemic control as compared to those who hadn't a history of diabetic complications. Contrary to this finding the studies done in India and at Tikur Anbessa Specialized Hospital, Ethiopia revealed that there were no significant associations between diabetic complications and poor glycemic control 13,30. This variation might be due to the method of glucose determination and ethnic factors, but the major cause of the diabetic complications is hyperglycemia which leads to advanced glycation end-product formation, oxidative stress, and atherosclerosis.

The result of the current study revealed that the odds of developing poor glycemic control were higher in study participants who had a lack of self-glucose monitoring when compared to the study participants who use self-glucose monitoring. This finding was similar to the studies conducted in Jimma and Tigray, Ethiopia 31,32. However, our finding was contrary to the studies reported from Saudi Arabia, Tanzania, and Gondar revealed that there were no significant associations between lack of self-glucose monitoring and poor glycemic control^27^,^33^,^34^. The possible reason for variation might be due to irregular use of glucometer at home and calibration problem of a glucometer.

The study participants who didn't perform physical exercise showed that the odds of developing poor glycemic control were higher when compared with those who perform physical exercise. This finding was in agreement with the studies done in Saudi Arabia 14, Nekemte Referral Hospital 26, and Gondar 18, this might be due to physical exercise increasing the consumption of glucose in muscle, decreasing visceral fat, increasing insulin sensitivity of receptors and reduce the circulation of the advanced glycation end product, unlike this studies, physical exercise was not significantly associated with the study conducted in Tanzania 34. This variation might be due to ethnic differences and not controlling confounders.

The respondents with a diabetic duration of ≥ 7 years were higher odds of poor glycemic control when compared to a duration of less than seven years. This finding was in agreement with studies conducted across Europe, Jordan, and India 13,24,35. This might be due to gradual impairment of insulin secretion by beta-cell as a result of the compensatory response of hyperglycemia, increase in insulin resistance and beta-cell of the pancreas may be damaged. Unlike these findings, the study conducted in Sudan revealed that there was no significant association between longer duration and poor glycemic control 36. This difference might be due to variations in the behavioral and socio-demographic characteristics.

Types of anti-diabetic medication were also a predictor of poor glycemic control in the current study. Patients on both oral hypoglycemic agents and insulin showed that the odds of having poor glycemic control were more likely lower when compared to the patients on insulin only. This finding was similar to the study done in Dessie, Ethiopia 37. This might be due to the synergistic effect of oral hypoglycemic agents and insulin because some oral hypoglycemic agents increase insulin secretion and tissue response to glucose.

Despite the fact that the rigorous effort was made to reduce or avoid the possible limitations of this study, our study results were interpreted under consideration of the following limitations. The nature of the cross-sectional study design didn't show a causality relationship of predictors and outcome variables. Not checking of conditions affect red blood cell turnover such as hemoglobinopathy. Using a self-response questionnaire to measure medication adherence is highly exposed to recall bias.

## Conclusion

The present study revealed that a high proportion of diabetic patients had poor glycemic control. Longer duration of DM, lack of self-glucose monitoring, having a history of diabetic complications, and dyslipidemia were significantly associated factors with poor glycemic control. The finding of the current study should be considered to develop strategic and timely interventions on identified risk factors to delay diabetic complications and improve the health outcome of diabetic patients. Further longitudinal studies should be conducted on the large population to identify predictors of poor glycemic control.

## Data sharing statements

All relevant data are within the manuscript and/or Supporting Information files.

## References

[1] Matthew C (2020). Riddle M. 6. Glycemic Targets. Standards of Medical Care in Diabetes.

[2] Hinge CR, Ingle SB, Adgaonkar BD (2018). Body Mass Index, Blood Pressure and Lipid profile in type 2 diabetes-Review.

[3] Longo DF, Longo D (2018). Harrisons principle of internal medicine.

[4] Guerin-dubourg A, Cournot M, Planesse C, Debussche X, Meilhac O, Rondeau P (2017). Association between Fluorescent Advanced Glycation End-Products and Vascular Complications in Type 2 Diabetic Patients. Biomed Res Int.

[5] Son C, Om I, Am A (2017). Vascular Complications in Diabetes Mellitus. Globa J Endocrinol Metab.

[6] Rhee SY, Kim YS (2018). Sulwon Lecture 2017 The Role of Advanced Glycation End Products in Diabetic Vascular Complications. Diabetes Metab J.

[7] Nam H (2019). Cho President 2017–2019s. Idf diabetes atlas.

[8] Naredi M, Jhavar D, Krishnan D (2017). Study of relationship between WBC count and diabetic complications. Int JAdvMed.

[9] WHO (2016). Global report on diabetes.

[10] Munson K (2019). Standard Operating Procedure. Gundersrn Heal Syst.

[11] Afsana A, Liaquat A, Karim N, Mohammed JA, Khurshid A, Dianna JM (2019). Glycaemic Control for People with Type 2 Diabetes Mellitus in Bangladesh. An urgent needfor optimization of management plan.

[12] Mellitus D, Woldu MA, Wami CD, Lenjisa JL, Tegegne GT, Tesafye G (2014). Endocrinology & Metabolic Syndrome Factors Associated with Poor Glycemic Control among Patients with Type 2. Endocrinol Metab Syndr.

[13] Borgharkar SS, Diab O, Care R (2019). Real-world evidence of glycemic control among patients with type 2 diabetes mellitus in India: the TIGHT study. BMG.

[14] Riyadh A, Alzaheb1 Abdullah H Altemani (2018). The prevalence and determinants of poor glycemic control among adults with type 2 diabetes mellitus in Saudi Arabia. Diabetes, Metab Syndr Obes Targets Ther.

[15] Duarte E M, Celestino R, Neves S, Conceic M, Almeida C De (2010). Glycemic control and its correlates in patients with diabetes in Venezuela: Results from a nationwide surveyõ Anto. diabetes Res clinical practice.

[16] Achila OO, Ghebretinsae M, Kidane A, Simon M, Makonen S, Rezene Y (2019). Factors Associated with Poor Glycemic and Lipid Levels in Ambulatory Diabetes Mellitus Type 2 Patients in Asmara, Eritrea: A Cross-Sectional Study. J Diabetes Res.

[17] Ababa A, Tekalegn Y, Addissie A, Kebede T, Ayele W (2018). Magnitude of glycemic control and its associated factors among patients with type 2 diabetes at Tikur Anbessa Specialized.

[18] Mesfin SM, abebe1 YB, alemayehu WS, alemu1 nebiyu (2015). Level of sustained glycemic control and associated factors among patients with diabetes mellitus in Ethiopia: a hospital-based cross-sectional study. Diabetes, Metab Syndr Obes.

[19] Ayano A, Yeshanew A (2015). The history and activities of wachemo university Nigis Elleni Mohammed memorial Hospital.

[20] Cheneke W, Suleman S, Yemane T, Abebe G (2016). Assessment of glycemic control using glycated hemoglobin among diabetic patients in Jimma University specialized hospital, Ethiopia. BMC Res Notes.

[21] Kubica A, Kosobucka A, Michalski P, Fabiszak T (2017). Self-reported questionnaires for assessment adherence to treatment in patients with cardiovascular diseases. viamedica.

[22] Consultation WHOE (2011). Waist Circumference and Waist-Hip Ratio Report of a WHO Expert Consultation.

[23] Alberti Professors Sir George, Zimmet Paul (2006). IDF, Metabolic Syndrome.

[24] Pablos-velasco P De, Parhofer KG, Bradley C, Eschw E, Maheux P, Wood I (2012). Current level of glycaemic control and its associated factors in patients with type 2 diabetes across Europe: Data from the PANORAMA study Current level of glycaemic control and its associated factors in patients with type 2 diabetes across Europe: data. PUBMED.

[25] Charles E (2014). Predictors of poor glycemic control in type 2 diabetic patients attending public hospitals in Dar es Salaam. Drug, Healthc Patient Saf 20146.

[26] Fekadu G, Bula K, Bayisa G, Turi E, Tolossa T, Kasaye HK (2019). Challenges And Factors Associated with Poor Glycemic Control Among Type 2 Diabetes Mellitus Patients at Nekemte Referral Hospital, Western.

[27] Biadgo B, Abebe M (2018). Glycemic control and diabetes complications among diabetes mellitus patients attending at University of Gondar Hospital, Northwest Ethiopia. Syndr Metab.

[28] Kakade AA, Mohanty IR, Rai S (2018). Assessment of factors associated with poor glycemic control among patients with Type II Diabetes mellitus. Integr Obes Diabetes Res.

[29] Daya R, Bayat Z, Raal FJ (2017). Prevalence and pattern of dyslipidaemia in type 2 diabetes mellitus patients at a tertiary care hospital Prevalence and pattern of dyslipidaemia in type 2 diabetes mellitus patients at a tertiary care hospital. J Endocrinol Metab Diabetes South Africa.

[30] Demoz GT, Gebremariam A, Yifter H, Alebachew M, Niriayo YL, Gebreslassie G (2019). Predictors of poor glycemic control among patients with type 2 diabetes on follow up care at a tertiary healthcare setting in Ethiopia. BMC Res Notes [Internet].

[31] Mamo Y, Bekele F, Nigussie T, Zewudie A (2019). Determinants of poor glycemic control among adult patients with type 2 diabetes mellitus in Jimma University Medical Center, Jimma zone, south west Ethiopia : a case control study. BMC Endocr Disord.

[32] Fseha B (2017). Journal of Diabetes & Metabolism Glycemic Control and it's Associated Factors in Type 2 Diabetic Patients. J Diabetes Metab.

[33] Altemani RAAAH (2018). The prevalence and determinants of poor glycemic control among adults with type 2 diabetes mellitus in Saudi Arabia. Diabetes, Metab Syndr Obes Targets Ther.

[34] Charles E (2014). Predictors of poor glycemic control in type 2 diabetic patients attending public hospitals in Dar es Salaam. Drug Healthc Patient Saf.

[35] Khattab M, Khader YS, Al-khawaldeh A, Ajlouni K (2010). Factors associated with poor glycemic control among patients with Type 2 diabetes. J Diabetes Complications.

[36] Omar SM, Musa IR, Osman OE, Adam I (2018). Assessment of glycemic control in type 2 diabetes in the Eastern Sudan. BMC Res Notes [Internet].

[37] Fiseha T, Alemayehu E, Kassahun W, Adamu A, Gebreweld A (2018). Factors associated with glycemic control among diabetic adult out patients in Northeast Ethiopia. BMC Res Notes [Internet].

